# Fabry Disease Therapy: State-of-the-Art and Current Challenges

**DOI:** 10.3390/ijms22010206

**Published:** 2020-12-28

**Authors:** Olga Azevedo, Miguel Fernandes Gago, Gabriel Miltenberger-Miltenyi, Nuno Sousa, Damião Cunha

**Affiliations:** 1 Cardiology Department, Reference Center on Lysosomal Storage Disorders, Hospital Senhora da Oliveira, 4835-044 Guimarães, Portugal; 2 Life and Health Sciences Research Institute (ICVS), School of Medicine, University of Minho, 4710-057 Braga, Portugal; miguelgago@hospitaldeguimaraes.min-saude.pt (M.F.G.); gmiltenyi@medicina.ulisboa.pt (G.M.-M.); njcsousa@med.uminho.pt (N.S.); damiaojlcunha@gmail.com (D.C.); 3 ICVS/3Bs PT Government Associate Laboratory, 4805-017 Braga/Guimarães, Portugal; 4 Neurology Department, Reference Center on Lysosomal Storage Disorders, Hospital Senhora da Oliveira, 4835-044 Guimarães, Portugal; 5 Genetics Department, Reference Center on Lysosomal Storage Disorders, Hospital Senhora da Oliveira, 4835-044 Guimarães, Portugal

**Keywords:** fabry disease, enzyme replacement therapy, agalsidase alfa, agalsidase beta, migalastat, pegunigalsidase alfa, moss-derived alfa galactosidase A, substrate reduction, mRNA, gene therapy

## Abstract

Fabry disease (FD) is a lysosomal storage disorder caused by mutations of the *GLA* gene that lead to a deficiency of the enzymatic activity of α-galactosidase A. Available therapies for FD include enzyme replacement therapy (ERT) (agalsidase alfa and agalsidase beta) and the chaperone migalastat. Despite the large body of literature published about ERT over the years, many issues remain unresolved, such as the optimal dose, the best timing to start therapy, and the clinical impact of anti-drug antibodies. Migalastat was recently approved for FD patients with amenable *GLA* mutations; however, recent studies have raised concerns that “in vitro” amenability may not always reflect “in vivo” amenability, and some findings on real-life studies have contrasted with the results of the pivotal clinical trials. Moreover, both FD specific therapies present limitations, and the attempt to correct the enzymatic deficiency, either by enzyme exogenous administration or enzyme stabilization with a chaperone, has not shown to be able to fully revert FD pathology and clinical manifestations. Therefore, several new therapies are under research, including new forms of ERT, substrate reduction therapy, mRNA therapy, and gene therapy. In this review, we provide an overview of the state-of-the-art on the currently approved and emerging new therapies for adult patients with FD.

## 1. Fabry Disease Overview

Fabry disease (FD) (OMIM 301500) is a rare X-linked lysosomal storage disorder caused by mutations in the *GLA* gene, leading to deficiency of the enzymatic activity of α-galactosidase A. Subsequent accumulation of globotriaosylceramide (GB3) and other neutral glycosphingolipids occurs in body fluids and lysosomes of cells throughout the body, including in those that are particularly relevant to disease pathology, such as in the heart (cardiomyocytes, conduction system cells, vascular endothelial, and smooth muscle cells and fibroblasts), kidney (podocytes, tubular, glomerular, mesangial, and interstitial cells), nervous system (neurons in autonomic and posterior root ganglia) and vascular endothelium and smooth muscle [1,2].

*GLA* mutations causing a virtually null enzymatic activity (<5% of the normal mean) are associated to severe and early onset classical phenotypes, while mutations leading to a residual enzymatic activity are associated to attenuated and late-onset phenotypes [1,2,3]. Classical phenotypes are characterized by early development, in childhood or adolescence, of acroparesthesias, neuropathic pain, hypohydrosis, heat, cold and exercise intolerance, cornea *verticillata*, angiokeratomas, gastrointestinal symptoms, and proteinuria. In adulthood, patients also suffer from sensorineural deafness and cardiac, renal, and cerebrovascular manifestations. In contrast, late-onset phenotypes are characterized by the development of cardiac, renal, and/or cerebrovascular manifestations in adulthood, and the phenotype may be dominated by the involvement of an organ, such as the heart or the kidneys [2,3,4,5]. Cardiac manifestations include left ventricular hypertrophy (LVH), heart failure, angina, dysrhythmias, cardiac conduction abnormalities, and sudden death. Renal involvement may lead to end-stage renal failure, and brain involvement is characterized by the development of brain white matter lesions (WML) and the occurrence of strokes or transient ischemic attacks (TIA) [2,3,4,5]. In this X-linked disorder, heterozygote females are not merely carriers, and their clinical spectrum ranges from asymptomatic to full-blown disease as severe as in affected males [6,7]. Ultimately, FD leads to a reduction of quality of life [8] and survival, with death being mainly driven by heart disease [9,10].

Available therapies for FD include enzyme replacement therapy (ERT) (agalsidase alfa and agalsidase beta) and the chaperone migalastat [11]. However, several new therapies are under research, including new forms of ERT, substrate reduction therapy, mRNA therapy, and gene therapy [12,13].

## 2. ERT

### 2.1. Efficacy and Safety of ERT

ERT with recombinant α-galactosidase A has been approved for clinical use since 2001. There are two commercially available preparations: agalsidase alfa, produced from human fibroblasts; and agalsidase beta, produced in Chinese hamster ovary (CHO) cells. Both are administered intravenously every other week (eow) at doses of 0.2 mg/kg and 1 mg/kg of body weight, respectively [11].

In males, agalsidase alfa has been demonstrated to decrease plasma GB3 [14,15,16] and lyso-GB3 [17] and urinary GB3 [14,16] levels; to decrease GB3 deposits in kidney endothelial cells [15]; to slow the decline of the estimated glomerular filtration rate (eGFR) [15,16]; to reduce/stabilize left ventricular (LV) mass [14,18,19] and wall thickness [19]; to improve/stabilize vestibular/auditory symptoms [20,21]; to improve nerve sensitivity [22], gastrointestinal symptoms [23], sweat function [23], pain [15], and pain-related quality of life [15].

In males, agalsidase beta has been shown to decrease plasma GB3 [24,25,26,27] and lyso-GB3 [26] and urinary GB3 [25] levels; to decrease GB3 deposits in different kidney cell types [24,25,28] and endothelial cells in skin [24,25]; to slow the decline of eGFR [25,29,30]; to reduce/stabilize LV mass [27,29,31,32] and wall thickness [27,32]; to improve nerve sensitivity [33], gastrointestinal symptoms [34], sweat function [33,35], pain [35], and quality of life [24,35].

In females, agalsidase alfa has been demonstrated to decrease plasma GB3 [36,37], plasma lyso-GB3 [17,26] and urine GB3 [36,37] (when pre-treatment values were elevated); to stabilize/decrease the decline of eGFR [19,36,37,38]; to decrease/stabilize LV mass [18,19,36,37] and wall thickness [19]; to improve exercise capacity [37]; to stabilize hearing loss and vestibular function [21]; and to improve quality of life [36].

In females, agalsidase beta has been shown to stabilize plasma GB3 (when pre-treatment values were normal) [26]; to decrease plasma lyso-GB3 [26] (when pre-treatment values were elevated); to stabilize eGFR [29]; to decrease/stabilize LV mass [29,32] and wall thickness [32]; and to improve quality of life [39].

Agalsidase alfa and beta are generally safe. The most common adverse events are infusion associated reactions (IAR), which are mild or moderate and tolerable in most cases [14,15,40,41] (Table 1).

### 2.2. Best Timing to Start ERT

According to the current recommendations, ERT should be initiated (i) in classic males at the age of 16 years regardless of symptomatic status, although it should be considered earlier, on an individual basis, since the age of 8–10 years old; and (ii) in late-onset males and in classic/late-onset females with signs/symptoms of FD (LVH, cardiac fibrosis, cardiac rhythm or conduction abnormality, microalbuminuria/proteinuria non-attributed to other causes, chronic kidney disease (eGFR <90 mL/min/1.73 m^2^), stroke or transient ischemic attack, neuropathic pain, gastrointestinal symptoms such as abdominal pain or diarrhoea, anhidrosis/hypohidrosis). In asymptomatic late-onset males and classic/late-onset females, ERT could be considered in the presence of moderate or severe GB3 deposits, podocyte foot process effacement, or glomerulosclerosis on kidney biopsy [11].

Early treatment has clearly been shown to achieve better outcomes.

Classic males who started ERT before the age of 25 years achieved greater reduction of plasma lyso-GB3 than the ones who started after that age [59]. Patients who started agalsidase beta at the age <30 years experienced a statistically significant decline in LV mass, while those who started it at the age ≥50 years suffered an increase in LV mass [31]. Likewise, patients who started agalsidase beta at the age <40 years had stable thickness of the interventricular septum and posterior wall over a period of 10 years, whereas patients who started it at the age ≥40 years significantly progressed over time [60]. Moreover, in patients without fibrosis, agalsidase beta resulted in a statistically significant decline of LV mass and improvement of exercise capacity and LV radial strain rate, while no effect was observed in patients with mild or severe fibrosis at the time of treatment initiation [61].

Additionally, the slope of eGFR decline was lower in patients who started agalsidase beta earlier since symptom onset and before the development of significant glomerulosclerosis and proteinuria [30,60].

Finally, the risk of clinical events was lower in patients who started agalsidase beta at younger age [62] and before severe organ damage [62,63]. Likewise, the risk of clinical events seemed to be lower in classic males who started ERT before the age of 25 years compared to the ones who started ERT later [59].

### 2.3. Optimal Dose of ERT

#### 2.3.1. Agalsidase Alfa vs. Agalsidase Beta at Licensed Dose

In classic males, the reduction in plasma lyso-Gb3 was significantly larger in patients treated for 1 year with agalsidase beta at the dose of 1.0 mg/kg than in patients treated with agalsidase alfa or beta at the dose of 0.2 mg/kg [26].

In another study, the reduction of plasma lyso-GB3 in classic males treated for 1 year with agalsidase beta 1 mg/Kg was greater than with agalsidase alfa 0.2 mg/Kg, and the same was observed in females and non-classic males. Additionally, a higher proportion of patients had a decrease in LV mass index when treated for 1 year with agalsidase beta than with agasidase alfa (79% vs. 62%). Nevertheless, no difference between agalsidase alfa and beta was found regarding eGFR or clinical events [64].

Another study showed that 19.4% of patients on agalsidase alfa 0.2 mg/Kg and 13.3% of patients on agalsidase beta 1 mg/Kg progressed, during the 59-month study duration, to a composite clinical endpoint consisting of renal events (development of end-stage renal disease or sustained decline in GFR of 50% or greater for more than 30 days and for which other causes besides FD had been excluded), cardiovascular events (pacemaker or other intracardiac device, coronary artery bypass grafting, valve replacement surgery, coronary angioplasty or stent, cardioversion, hospitalization or emergency room visit for unstable angina/acute coronary syndrome, myocardial infarction, congestive heart failure, tachy- or brady-arrhythmia, heart block, cardiac arrest), cerebrovascular events (TIA or stroke documented by a physician or acute hearing loss), or death. However, these differences were not significant due to limited power [65].

#### 2.3.2. Agalsidase Beta at Reduced Dose

The dose reduction of agalsidase beta from 1 mg/Kg eow to 0.3 mg/Kg eow for 18 months allowed for maintaining the clearance of GB3 deposits in the kidney interstitial capillary endothelium in 90% of the patients; and the clearance/reduction of GB3 deposits in other renal cells and superficial dermal capillary endothelium in only 70% of the patients. Additionally, urinary GB3 increased significantly after dose reduction [25].

In another study, the dose reduction of agalsidase beta from 1 mg/Kg eow to 0.5 mg/Kg per month for 1 year led to a significant increase of plasma lyso-GB3 in males, but no differences were seen on clinical events, Mainz Severity Score Index (MSSI), pain, eGFR, or LV mass [66].

In the Fabry registry, the dose reduction of agalsidase beta resulted in lower self-reported energy levels in males, but no difference in MSSI, DS3, Brief Pain Inventory (BPI), or SF-36 scores [67].

#### 2.3.3. Switch between Therapies

Patients under agalsidase beta 1 mg/kg eow, who switched directly to agalsidase alfa 0.2 mg/Kg eow or suffered a dose reduction to agalsidase beta 0.3–0.5 mg/kg eow and then switched to agalsidase alfa 0.2 mg/Kg eow, showed a greater eGFR decline and an increase of MSSI, GI symptoms [68,69], and pain [68], although clinical events remained stable for 2 years [69].

In three young classic males under agalsidase beta 1 mg/kg eow for 5 years, there was complete clearance of GB3 from mesangial and endothelial cells and partial clearance from podocytes. Three years after the switch to agalsidase alfa 0.2 mg/Kg eow, there was reaccumulation of GB3 in podocytes. One patient switched back to agalsidase beta 1 mg/Kg eow and two years later again showed reduction of GB3 in podocytes. Additionally, pain and GI symptoms worsened in all three patients following the switch from agalsidase beta to alfa [70].

However, in another study, patients under agalsidase beta 1 mg/kg eow who switched to agalsidase alfa 0.2 mg/Kg eow did not show any differences on plasma lyso-GB3 or GB3, eGFR, proteinuria, or LV mass index for 2 years [17].

#### 2.3.4. Agalsidase Alfa at Increased Dose

A study comparing patients under agalsidase alfa 0.2 mg/Kg eow and 0.2 mg/kg weekly for one year did not find any significant differences in plasma GB3, LV mass, eGFR, albuminuria, 6-min walk test, or quality of life. Exploratory analyses of patients under 0.4 mg/kg weekly did not find any differences either [71].

Additionally, a study comparing patients under agalsidase alfa 0.1 mg/kg weekly, and 0.2 mg/Kg eow and 0.2 mg/Kg weekly for 4 weeks, did not find any significant differences on plasma GB3, quality of life, or pain, although there was a trend for a higher sweat volume and a lower urinary GB3 with the weekly dose of 0.2 mg/Kg [72].

Conversely, in patients with progressive decline of renal function despite agalsidase alfa 0.2 mg/kg eow for 2–4 years, the switch to 0.2 mg/Kg weekly significantly decreased the annual slope of eGFR during a 10 year-study, significantly delaying the time to end-stage renal disease [73,74].

### 2.4. Limitations of ERT

ERT presents several potential limitations: (i) It has limited tissue penetration; (ii) it does not pass the blood-brain barrier; (iii) it may induce infusion adverse reactions; (iv) it may induce the production of anti-drug antibodies with neutralizing effect, reducing the efficacy of the therapy; (v) it is a lifelong therapy requiring intravenous administration every 2 weeks; and (vi) it is associated to a high cost (Table 2).

#### 2.4.1. Limited Tissue Penetration

The inefficient biodistribution of the exogenous recombinant enzyme may contribute to the limited efficacy of ERT. While most of the administered recombinant enzyme is taken up by the liver, cardiomyocytes and podocytes, despite being severely affected by FD, only take up few amounts of recombinant enzyme, which might contribute to the limited clearance of GB3 deposits in these cells [75,76].

Mannose 6-phosphate (M6P) mediated endocytosis has been considered the main mechanism of uptake of recombinant α-galactosidase A. However, the rate of cellular uptake among different tissues depends on the pattern of glycosylation and phosphorylation of the enzyme, which is different between agalsidase alfa and agalsidase beta, because post-translational protein modifications are specific to the species [77].

Nevertheless, blocking the M6P receptor inhibited the uptake of recombinant α-galactosidase A in fibroblasts, but not in endothelial cells [78]. Moreover, in podocytes, enzyme uptake is known to be mediated by M6P, megalin, and sortilin receptors, but blocking all three receptors only inhibited the uptake of recombinant enzyme by 39% [79]. These results suggest the existence of additional uptake mechanisms, which might explain the existence of different biodistribution profiles among tissues.

#### 2.4.2. No Crossing of the Blood-Brain Barrier

Stroke and TIA in Fabry patients have been attributed to cardioembolism or vascular involvement by the disease; however, the pathophysiology of brain WML is probably more complex. GB3 deposits within the endothelial and smooth muscle cells of the small and medium-size brain arteries may decrease vascular compliance and impair autoregulation of cerebral perfusion and lead to endothelial dysfunction, increase of pro-thrombotic/pro-inflammatory cytokines, and upregulation of the renin–angiotensin system, which may result in ischemic events [80]. On the other hand, GB3 deposits were also found in brain neurons and glia [81,82], although a clinical correlate remains to be established [83]. Glial dysfunction and neuroinflammation may also contribute to the development of brain WML [80]. Yet, their clinical impact is not fully understood.

None of the recombinant enzyme preparations can pass the blood–brain barrier. The effect of ERT on the development or progression of brain WML remains to be elucidated. During a 2-year follow-up of six patients under agalsidase alfa, brain WML remained stable in three patients, worsened in one, fluctuated in one, and improved in one patient; during this time, some brain WML disappeared and others appeared on brain MRI [84]. During a mean follow-up of 27 months, agalsidase beta seemed to stabilize brain WML on MRI compared to placebo, as a statistically significant greater proportion of younger patients (≤50 years) under agalsidase beta had stable WML compared with younger patients under placebo (44% vs. 31%) [85].

#### 2.4.3. Infusion-Associated Reactions

ERT leads to IAR, which occur mostly in the first 13 infusions and are mostly limited to fever and chills [86], although life-threatening reactions have also been reported [87]. IAR occur in 14% of patients under agalsidase alfa [45] and 67% under agalsidase beta [46]; and the risk of IAR seems higher in males with nonsense or null mutations (CRIM negative) [86] and in patients with an anti-agalsidase IgG antibody–positive status [86,88].

#### 2.4.4. Anti-Drug Antibodies

Anti-drug antibodies develop in the first 3–6 months of ERT [86,89], mostly in classic males [90]. Anti-drug antibodies have been reported in 91% of males treated with agalsidase beta [57] and 20% of males treated with agalsidase alfa [14], although no significant difference in their formation has been reported when the same dosage (0.2 mg/kg every 2 weeks) was used for both drugs at ERT initiation [91]. Antibodies have shown in vitro cross-reactivity to both agalsidase alfa and beta [89].

IgG antibodies measured by ELISA mediate a neutralizing activity [89]. Neutralizing anti-drug antibodies develop in about 40% of all ERT-treated males [86,88] and mostly in patients treated with agalsidase beta [64]. Their formation seems to be irreversible, and the majority of patients positive for neutralizing anti-drug antibodies remain as so over 10 years [64,88,91].

These anti-drug antibodies bind and neutralize ERT in plasma and lead to activation of macrophages that internalize the ERT-antibody complexes, decreasing the cellular uptake of ERT [89].

Neutralizing anti-drug antibodies may attenuate ERT efficacy. Anti-drug antibodies have been associated to higher frequency of GB3 deposits in endothelial cells of the skin [92] and higher plasma lyso-GB3 [64,88,91,93] and urinary GB3 [89,91]. Although previous reports have suggested that anti-drug antibodies had no effect on the time to first clinical event or eGFR slope [92], other studies have shown that they were associated to worse renal function [88,93] and higher LV mass, disease severity scores, and frequency of symptoms [88].

Increasing the ERT dose in patients with established anti-drug antibodies may saturate them and allow for the excess enzyme to perform its catalytic function, thereby attenuating the negative effect of anti-drug antibodies in plasma lyso-Gb3, eGFR, and LV wall thickness [94].

## 3. Migalastat

### 3.1. Efficacy and Safety of Migalastat

Migalastat is a first-in-class pharmacological chaperone therapy for FD. It is a low molecular weight iminosugar analogue of the terminal galactose residue of GB3 that selectively and reversibly binds to the active site of amenable mutant forms of α-galactosidase A, thereby stabilizing the enzyme, preventing its retention and degradation in the endoplasmic reticulum, and facilitating its trafficking to lysosomes. Once in lysosomes, migalastat dissociates from α-galactosidase A, due to the more acidic pH and higher concentration of substrates, allowing the enzyme to exert its activity on GB3 [42,43,44].

Migalastat is an oral drug administered at the dosage of 123 mg once every other day, which has been approved in 2016 by the European Medicines Agency for the treatment of FD patients aged ≥16 years, with eGFR ≥30 mL/min/1.73 m^2^ and amenable *GLA* mutations [47].

In the FACETS trial, in the modified-intention to treat population (i.e., ERT-naïve patients with migalastat-amenable *GLA* mutations), migalastat, compared to placebo, significantly reduced the plasma lyso-GB3 and the mean number of GB3 inclusions/kidney interstitial capillary at 6 months. There were no significant differences in baseline levels or changes from baseline to month 6 between the groups regarding eGFR, mGFR, 24 h-urine protein excretion, and 24 h urinary GB3. At 24 months of migalastat therapy (i.e., after 18 months of migalastat in patients who switched from placebo or 24 months of continuous migalastat), there was a significant reduction of the mean LV mass index compared to the baseline [48]. An improvement in diarrhea was also found after 6 months of migalastat compared to placebo, and this benefit was sustained at 24 months [48,56]. Those results were also confirmed in the subgroup of classic males [95]. Migalastat treatment was also demonstrated to decrease the mean total GB3 inclusion volume per podocyte in renal biopsies from baseline to 6 months; and this reduction correlated with the reduction in mean podocyte volume [50].

In the ATTRACT trial, in ERT-experienced patients with amenable *GLA* mutations who were randomized to switch to migalastat or continue ERT, renal function and plasma lyso-GB3 levels were maintained during 18 months of migalastat or ERT. Migalastat significantly reduced the mean LV mass index at 18 months, and changes on LV mass index correlated with changes in the thickness of the interventricular septum and not the posterior wall. No significant change of the mean LV mass index was found in the ERT group; however, the randomization ratio of migalastat to ERT of 1.5 to 1, the existence of non-amenable mutations in the initial cohort, and the higher discontinuation rate on the ERT group resulted in a small number of evaluable patients under ERT at the end of the study (16 patients), which may have influenced the results regarding ERT [51]. In the open-label extension study, a significant decrease of LV mass index was found after 30 months of migalastat in patients with LVH at baseline [52].

In real world conditions, Muntze et al. reported one patient with an improvement of LV mass, LGE, troponin, and NT-proBNP after 12 months of treatment with migalastat [53]. Later, the same authors showed that, in 14 patients treated with migalastat for 1 year, LV mass index significantly decreased, while plasma lyso-GB3 significantly decreased in naïve patients and remained stable in patients switched from ERT. However, eGFR significantly worsened, a finding that contrasted to the results in the pivotal clinical trials. The authors hypothesized that these results could have been partly explained by the simultaneous initiation of angiotensin converting enzyme inhibitors and underlined the need for further studies with longer follow-up of the renal function [49].

In a larger study including 59 patients, treatment of previously ERT-treated and untreated FD patients with migalastat for 12 months was generally safe and also resulted in a significant decrease of LV mass index. However, plasma lyso-GB3 levels were stable both in males and females, irrespective of previous treatment regimen; and increasing plasma lyso-GB3 was noted in a few males and females, some of whom carried *GLA* mutations whose amenability to migalastat has been questioned. Moreover, eGFR continued to decline under migalastat in both males and females. The loss of renal function did not seem to be explained by a more severe renal impairment and disease load at baseline. Instead, higher decline of eGFR was more common in patients with a systolic blood pressure below 120 mmHg. Additionally, females with *GLA* mutations, classified as non-amenable by the in-house assay based on *GLA*-knockout HEK cells, had higher decline of eGFR. This study recommended to avoid systolic blood pressure values below 120 mmHg and alerted that “in vitro” amenability may not always reflect “in vivo” amenability to migalastat, emphasizing the importance of monitoring clinical response to therapy [54].

In a smaller study of seven FD males, who switched from ERT to migalastat, a significant decrease of LV mass index was also observed after 1 year of treatment with migalastat, while plasma lyso-GB3 remained stable. Unlike previous real-life studies, eGFR was stable and proteinuria significantly decreased [55]. Therefore, further studies are needed to understand the discrepant results of migalastat on renal function.

Migalastat is generally well tolerated, with headache and nasopharyngitis being the most common side effects [48,51]. Transient and fully reversible infertility was also found in male rats, but its occurrence in humans remains to be established [47] (Table 1).

### 3.2. “In Vitro” and “In Vivo” Amenability to Migalastat

The amenability of *GLA* mutations to migalastat is determined by a good laboratory practice (GLP) “in vitro” pharmacogenetics assay, which has been clinically validated. This assay uses human embryonic kidney (HEK) 293 cells that have been transfected with individual *GLA*-containing DNA plasmids to measure increases in α-galactosidase A activity in response to migalastat. According to Benjamin et al., *GLA* mutations that do not qualify for testing include large deletions, insertions, truncations, frameshift mutations, and splicing mutations; these mutations are classified as non-amenable. *GLA* mutations that qualify for testing include missense mutations, nonsense mutations near the carboxyl terminus, small insertions and deletions that maintain reading frame, and complex mutations comprising two or more of these types of mutations on a single *GLA* allele. Migalastat-amenable mutations are defined as *GLA* mutations that translate to mutant forms of α-galactosidase A that, in the presence of 10 μmol/L migalastat, display an increase of the enzymatic activity ≥1.2-fold over baseline and an absolute increase ≥3% of the enzymatic activity quantified as a percentage of the enzymatic activity of the wild-type α-galactosidase A [96]. It is estimated that 35–50% of FD patients have mutations that are amenable to migalastat [51].

However, recent studies have reported that migalastat was associated to an insufficient increase of the enzymatic activity of α-galactosidase A and increasing values of plasma lyso-GB3 in patients with certain *GLA* gene mutations classified as amenable based on the “in vitro” GLP-HEK assay, thereby raising concerns that “in vitro” amenability may not always reflect “in vivo” amenability [54,97,98]. Hence, serial measurement of the enzymatic activity of α-galactosidase A in leukocytes and monitoring of the clinical response are mandatory in order to assess “in vivo” amenability to migalastat.

### 3.3. Limitations and Potential Advantages of Migalastat

Migalastat is a therapeutic option only for patients with amenable *GLA* mutations [47,48,51,96]. Due to the lack of data, migalastat is not recommended in patients aged ≥75 or <16 years, pregnant or breastfeeding, or with severe renal impairment (eGFR <30 mL/min/1.73 m^2^) [47,48,51].

Migalastat presents several potential advantages: (i) It is an oral therapy, thereby eliminating the requirement for lifelong intravenous infusions; (ii) it is a non-immunogenic molecule, avoiding antibody-related tolerability issues of ERT; (iii) it allows for sustained and stable enzyme levels that more closely mimic those of endogenous wild-type enzymes, whereas ERT leads to fluctuating and intermittent enzymatic activity; (iv) as a small molecule, it is likely to have enhanced cellular and tissue distribution; (v) and potential to cross the blood-brain barrier [58], as suggested by the finding of increased α-galactosidase A activity and reduced GB3 levels in the brain of Fabry transgenic mice [99] (Table 2).

### 3.4. Emerging New FD Therapies

Current therapies have not shown to be able to fully revert FD pathology and clinical manifestations. Therefore, several new therapies are under research, including new forms of ERT (pegunigalsidase alfa, moss-derived α-galactosidase A), substrate reduction therapy (lucerastat, venglustat), mRNA therapy, and genetic therapy [12,13] (Table 3).

Pegunigalsidase alfa, a chemically modified α-galactosidase A enzyme produced in a tobacco plant cell based ProCellEX system, is constituted by two subunits of α-galactosidase A covalently bound by a chain of polyethylene glycol (PEG), which increases its stability and reduces its clearance, thereby extending its plasma half-life [100,101,102] and allowing a monthly infusion [102]. Being plant-derived, this enzyme does not display M6P on their surface glycans [100], which suggests an alternative mechanism of cell uptake and may result in a different biodistribution profile from agalsidase alfa and agalsidase beta. This enzyme has already been demonstrated (i) to decrease plasma lyso-Gb3 levels and peritubular capillary Gb3 deposits in the kidney; (ii) to improve the BPI score, gastrointestinal symptoms, and MSSI; (iii) and to maintain eGFR, proteinuria and LV mass on MRI with no “de novo” cardiac fibrosis [101]. Studies to further test its efficacy are ongoing [102,103]. Anti-drug antibodies occurred in 19% of cases and became negative after 1 year, suggesting induction of immune tolerance [101], but its effect on the immune system is not yet completely clear [13]. Immune tolerance may occur as a result of the extended half-life and higher exposure of the immune system to the enzyme; or because pegylation may mask the enzyme to the immune system. However, concerns about immunogenicity have also been raised, because of the different glycosylation pattern of a plant-derived protein and the PEG component. Of note, the extended half-life may also interfere with the laboratory assays for anti-drug antibodies detection, as the circulating enzyme at the sampling time may bind antibodies and prevent their detection [13].

Moss-derived α-galactosidase A is produced in the moss *Phycomitrella patens* and is a glycoengineered variant devoid of α-1,3-fucose and β-1,2-xylose residues on its N-glycans that are plant-specific and may elicit immunogenic response in mammals [104]. Being plant-derived, this enzyme does not display M6P on their surface glycans; instead, it carries >90% mannose-terminated glycans [104]. This enzyme has a shorter half-life, which might be due to a higher cellular uptake via the mannose receptors [104,105], and has shown to reduce urinary Gb3 in a higher proportion than previously reported for α-galactosidase A produced in mammalian cells [105].

The glucosylceramide synthetase (GCS) inhibitors, such as venglustat and lucerastat, block the enzyme catalyzing the first step of glycosphingolipid biosynthesis, reducing glucosylceramide and Gb3 [13]. In patients with residual enzyme activity, GCS inhibitors might be sufficient to reduce the synthesis of GB3 to a level manageable by the residual enzyme activity, while in classic patients, with minimal to no residual enzyme activity, they may not be sufficient in monotherapy, but can still be useful in combination with ERT [13]. Venglustat, also known as ibiglustat, has shown to reduce GB3 deposits in tissues (including in brain) and plasma GB3 and lyso-GB3 in mice, this effect being more pronounced when added to ERT, except in the brain, where ERT had no effect [106]. Preliminary data also showed the reduction of Gb3 from superficial skin capillary endothelium and plasma lyso-Gb3 in treatment-naïve Fabry patients [13]. Lucerastat added to ERT resulted in a reduction of plasma GB3, while no reduction was seen in patients under ERT alone; and in stabilization of renal and cardiac parameters at 12 weeks [109]. Despite the clear advantages of oral administration, absence of anti-drug antibodies, and the possibility of passing the blood-brain barrier, caution in dosing should be taken, because the complete block of a single enzymatic reaction could potentially disrupt the cell homeostasis [13].

mRNA therapy, encapsulated in lipid nanoparticles, primarily targets hepatocytes in which the enzyme is produced and secreted into the circulation [13]. In mice and non-human primates, mRNA therapy has been shown to reduce GB3 and lyso-GB3 in heart and kidney [107,108] and plasma [108], and this effect was maintained for up to six weeks after infusion, suggesting that it could have the advantage of a larger interval between infusions [107,108]. Unlike ERT, mRNA therapy carries the advantage of using the endogenous protein translation system to ensure proper folding, glycosylation, and intracellular trafficking of α-galactosidase A; and, unlike DNA-based therapy, it does not carry a risk for insertional mutagenesis [13].

Gene therapy, using viral or non-viral vectors, may introduce a correct version of *GLA* gene, through “in vivo” or “ex vivo” technology [12]. The first FD patients have been treated in phase I and II clinical trials using an “ex vivo” approach, in which hematopoietic stem cells of the patient were recruited, transfected using lentiviruses, and re-administered to the patient. The main challenge is to target all affected cell types and tissues. Additionally, it is unclear whether classic males will develop antibodies against the expressed enzyme [13].

## 4. Conclusions

Despite ERT has demonstrated efficacy and safety on the treatment of FD, there are still pending questions about the best regimen and timing to start therapy as well as limitations, such as the limited tissue penetration, the immunogenicity issues, and the inconvenience of lifelong biweekly intravenous administrations.

Migalastat, the first-in-class pharmacological chaperone therapy for FD, has proven to be safe and efficacious in patients with amenable *GLA* mutations. Being an oral non-immunogenic drug, migalastat has overcome some of the limitations of ERT; however, clinical evidence is still growing, amenability issues have been reported, and its clinical use is limited to about 35–50% of FD patients.

As both therapies have been unable to fully revert FD pathology and clinical manifestations, this remains an enthusiastic field of investigation, with several new emerging therapies under research and development, such as new forms of ERT, substrate reduction therapy, and mRNA and gene therapies. So far, it remains unclear if optimal treatment lies on a single therapy or combination of therapies; and whether other therapeutic strategies, beyond the correction of the enzymatic defect alone, will be needed to avoid or revert organ damage in FD.

## Figures and Tables

**Table 1:** Currently approved therapies for adult patients with FD.

	Agalsidase Alfa	Agalsidase Beta	Migalastat
**Definition**	Recombinant α-galactosidase A produced from human fibroblasts [11]	Recombinant α-galactosidase A produced from Chinese hamster ovary (CHO) cells [11]	Low molecular weight iminosugar analogue of the terminal galactose residue of GB3 [42,43,44]
**Mechanism of action**	Enzyme replacement therapy [11]	Enzyme replacement therapy [11]	Pharmacological chaperone that selectively and reversibly binds to the active site of amenable mutant forms of α-galactosidase A, stabilizing it, preventing its retention in the ER, and enabling its trafficking to the lysosomes [42,43,44]
**Administration route**	Intravenous [45]	Intravenous [46]	Oral [47]
**Dose and frequency of administration**	0.2 mg/Kg every other week [45]	1 mg/Kg every other week [46]	123 mg once every other day [47]
**Efficacy**	*Males* ▪ Decreases plasma GB3 [14,15,16] and lyso-GB3 [17] and urinary GB3 [14,16] levels ▪ Decreases GB3 deposits in kidney endothelial cells [15] ▪ Slows the decline of eGFR [15,16] ▪ Reduces/stabilizes LV mass [14,18,19] and wall thickness [19] ▪ Improves/stabilizes vestibular/auditory symptoms [20,21] ▪ Improves nerve sensitivity [22], gastrointestinal symptoms [23], sweat function [23], pain [15] and pain-related quality of life [15] *Females* ▪ Decreases plasma GB3 [36,37], plasma lyso-GB3 [17,26], and urine GB3 [36,37] (when pre-treatment values were elevated) ▪ Stabilizes/decreases the decline of eGFR [19,36,37,38] ▪ Decreases/stabilizes LV mass [18,19,36,37] and wall thickness [19] ▪ Improves exercise capacity [37] ▪ Stabilizes hearing loss and vestibular function [21] ▪ Improves quality of life [36]	*Males* ▪ Decreases plasma GB3 [24,25,26,27] and lyso-GB3 [26] and urinary GB3 [25] levels ▪ Decreases GB3 deposits in different kidney cell types [24,25,28] and endothelial cells in skin [24,25] ▪ Slows the decline of eGFR [25,29,30] ▪ Reduces/stabilizes LV mass [27,29,31,32] and wall thickness [27,32] ▪ Improves nerve sensitivity [33], gastrointestinal symptoms [34], sweat function [33,35], pain [35], and quality of life [24,35] *Females* ▪ Stabilizes plasma GB3 (when pre-treatment values were normal) [26] ▪ Decreases plasma lyso-GB3 [26] (when pre-treatment values were elevated) ▪ Stabilizes eGFR [29] ▪ Decreases/stabilizes LV mass [29,32] and wall thickness [32] ▪ Improves quality of life [39]	▪ Decreases plasma lyso-GB3 [48,49] ▪ Decreases mean number of GB3 inclusions/kidney interstitial capillary [48] and mean total GB3 inclusion volume per podocyte [50] ▪ Reduces mean LV mass index [48,49,51,52,53,54,55] ▪ Improves diarrhoea [48,56] ▪ No change in eGFR, mGFR, 24 h-urine protein excretion and 24 h urinary GB3 in pivotal clinical trials [48,51]; however, decline of eGFR was reported in some real-life studies [49,54]
**Safety**	▪ IAR are the most common side effects (mainly mild, such as fever and chills) [14,15] ▪ Hypersensitivity reactions are rare [45] ▪ Anti-drug antibodies in 20% of treated males [14]	▪ IAR are the most common side effects (mainly mild, such as fever and chills) [40,41] ▪ Hypersensitivity reactions are rare [46] ▪ Anti-drug antibodies in 91% of treated males [57]	▪ Headache and nasopharyngitis are the most common side effects [48,51] ▪ No immunogenicity issues [58]

eGFR, estimated glomerular filtration rate; ER, endoplasmic reticulum; IAR, infusion associated reactions; LV, left ventricular; mGFR, measured glomerular filtration rate.

**Table 2:** Advantages and limitations of current therapies of FD.

	Advantages	Limitations
**ERT**	▪ Large body of evidence supporting its efficacy and safety ▪ Long clinical experience, being commercially available since 2001	▪ Incomplete reversion of FD pathology and clinical manifestations ▪ Limited tissue penetration ▪ No crossing of the blood–brain barrier ▪ Infusion adverse reactions ▪ Anti-drug antibodies with neutralizing effect may reduce the efficacy of ERT ▪ Lifelong therapy requiring intravenous administration every 2 weeks ▪ High cost
**Migalastat**	▪ Oral administration ▪ Small molecule, likely to have enhanced cellular and tissue distribution ▪ Sustained and stable enzyme levels ▪ Non-immunogenic molecule ▪ Favorable safety profile	▪ Therapeutic option only for patients with amenable *GLA* mutations ▪ “In Vitro” amenability may not always reflect “in vivo” amenability ▪ Not recommended in patients aged ≥75 or <16 years, pregnant or breastfeeding, or with severe renal impairment (eGFR <30 mL/min/1.73 m^2^) due to lack of data ▪ Incomplete reversion of FD pathology and clinical manifestations ▪ High cost

eGFR, estimated glomerular filtration rate; ERT, enzyme replacement therapy; FD, Fabry disease.

**Table 3:** Potential advantages and limitations of emerging therapies of FD.

	Potential Advantages	Potential Limitations
**Pegunigalsidase alfa**	▪ Higher plasma half-life [100,101,102], allowing a monthly infusion [102]	▪ Anti-drug antibodies in 19% of cases [101] ▪ Plant-derived protein with a different glycosylation pattern—possible immunogenicity issues? [100] ▪ Unclear effect on the immune system [13] ▪ No crossing of the blood–brain barrier [13] ▪ Lifelong therapy requiring intravenous administration [13]
**Moss-derived α-galactosidase A**	▪ Higher cellular uptake via the mannose receptors [104,105]	▪ Plant-derived protein with a different glycosylation pattern—possible immunogenicity issues? [104] ▪ Unclear effect on the immune system [104] ▪ No crossing of the blood–brain barrier [13] ▪ Lifelong therapy requiring intravenous administration [13]
**Substrate reduction therapy**	▪ Oral administration [13] ▪ Non-immunogenic [13] ▪ Possible crossing of the blood-brain barrier [106]	▪ Complete block of a single enzymatic reaction could potentially disrupt the cell homeostasis [13] ▪ May not be sufficient as monotherapy for patients with minimal/no residual enzymatic activity [13]
**mRNA therapy**	▪ Potential for a larger interval between infusions [107,108] ▪ Uses endogenous protein translation system to ensure proper folding, glycosylation, and intracellular trafficking of α-galactosidase A [13] ▪ No risk of insertional mutagenesis [13]	▪ Primarily targets hepatocytes [13] ▪ Unclear effect on immune system in classic males [13]
**Gene therapy**	▪ Introduces a correct version of the *GLA* gene [12] ▪ Uses endogenous protein translation system to ensure proper folding, glycosylation, and intracellular trafficking of α-galactosidase A [13]	▪ Targeting all affected cell types and tissues is a challenge [13] ▪ Risk of insertional mutagenesis [13] ▪ Unclear effect on immune system in classic males [13]
